# Hodgkin Lymphoma Presenting as Cardiac Tamponade in a Young Female

**DOI:** 10.1155/2024/5597263

**Published:** 2024-07-08

**Authors:** Georgia Kaiafa, Stylianos Daios, Stavroula Bountola, Triantafyllia Koletsa, Michail Makris, Charikleia Chatzikosma, Antonia Loukousia, Vasileios Perifanis, Antonios Ziakas, Christos Savopoulos

**Affiliations:** ^1^ First Propedeutic Department of Internal Medicine AHEPA University Hospital of Thessaloniki Aristotle University of Thessaloniki 54636, Thessaloniki, Greece; ^2^ First Department of Cardiology AHEPA University Hospital School of Medicine Faculty of Health Sciences Aristotle University of Thessaloniki, St. Kyriakidi 1 54636, Thessaloniki, Greece; ^3^ Department of Pathology School of Medicine Aristotle University of Thessaloniki, Thessaloniki, Greece

## Abstract

Hodgkin lymphoma (HL) is an uncommon malignancy that is characterized by Hodgkin or Reed–Sternberg cells. Cardiac implications of HL remain one of the least investigated subjects. There are few case reports in the literature of cardiac tamponade in HL patients. We describe a case of a 21-year-old female patient who presented with cardiac tamponade as an initial presentation of HL. Any pericardial effusion significant for tamponade requires immediate drainage and fluid analysis for thorough investigation. Prompt identification and timely intervention are crucial in effectively addressing these complex situations. Therefore, clinicians should maintain heightened awareness in such cases.

## 1. Introduction

In the vast landscape of medical complications associated with Hodgkin lymphoma (HL), pericardial effusion stands out, despite being observed in 5% of the patients [1]. Notably, only few patients previously diagnosed with HL-manifesting pericardial effusion have been reported in the literature, while on the contrary, many of them remain asymptomatic [1–3]. In addition, few non-HL cases present with cardiac tamponade as an inaugural symptom [4]. Our intention is to shed light on a unique case where a patient's primary implication of HL was marked by symptomatic pericardial effusion leading to cardiac tamponade.

Pericardium is a double-walled sac containing the heart and the roots of the great vessels. It has two layers, an outer layer made of strong inelastic connective tissue (fibrous pericardium) and an inner layer made of the serous membrane (serous pericardium). It contains 20 to 60 mL of ultrafiltrate and is inherently rigid [5]. In case of an acute cardiac tamponade, the pericardium rapidly accumulates approximately 200 to 300 mL of fluid, leading to a more abrupt onset of symptoms [5]. Conversely, in subacute cardiac tamponade, the pericardial sac gradually accumulates up to 1 to 2 L of fluid, resulting in a more insidious development of symptoms [5].

## 2. Case Presentation

A 21-year-old female student, without a previous medical history, presented to the emergency department with chest pain and shortness of breath during the last week.

According to her thorough examination in the emergency department, her initial blood pressure was 98/69 mmHg, her pulse rate was 115 beats/min, her respiratory rate was 23 breaths/min, and her oxygen saturation was 97% with a nasal cannula. She demonstrated elevated and distended jugular venous pressure with pulsus paradoxus and muffled heart sounds. Lung auscultation revealed reduced breath sounds at the bilateral lower zones.

**Figure 1:**
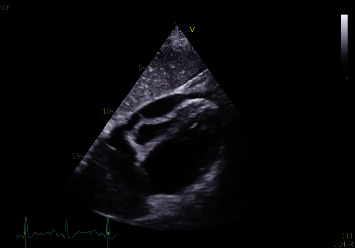
Presence of a pericardial effusion with right ventricular collapse during diastole.

**Figure 2:**
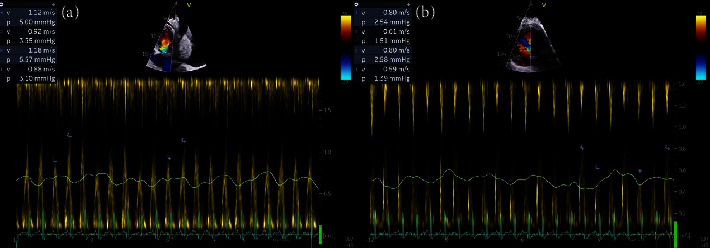
Respiratory variation of mitral (a) and tricuspid (b) inflow velocities.

Electrocardiogram demonstrated sinus tachycardia with low QRS voltage and electric alternans, while the transthoracic echocardiography indicated the presence of a pericardial effusion with right ventricular collapse during diastole (Figure 1) and respiratory variation of mitral and tricuspid inflow velocities (Figure 2). Meanwhile, the chest X-ray of the patient indicated an enlargement of the mediastinum.

The patient was admitted to the Coronary Care Unit, emergency pericardiocentesis was performed, and 600 ml of pericardial fluid was drained. Routine and microscopic findings of the pericardial fluid showed white blood cells: 56.388 Κ/*μ*L (mononuclear cells: 98% and neutrophils: 2%) without the presence of a microorganism after Gram‐stain and acid-fast bacilli stain. Biochemical examination of the pericardial fluid showed total protein, 4.5 g/dl (reference range, 1.5–4.5 g/dl); glucose, 61 mg/dl (reference range, 74−100 mg/dl); and lactate dehydrogenase (LDH), 411 U/L.

**Figure 3:**
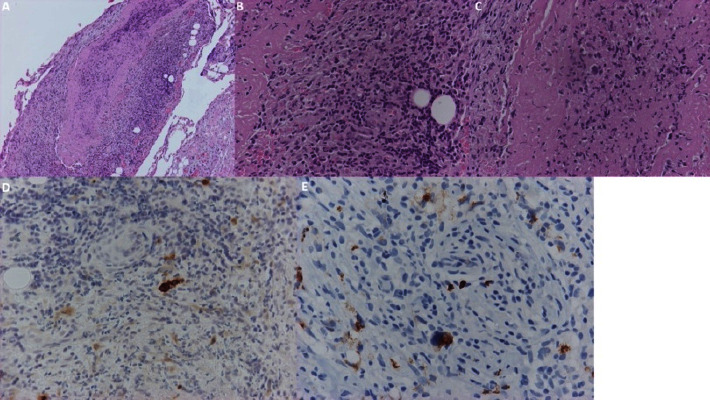
(A) Pericardium with fibrin and infiltration by inflammatory cells (HE ×100). (B) Neoplastic Hodgkin and Reed–Sternberg (HRS) cells intermingled with inflammatory cells (HE ×400). (C) A large Reed–Sternberg cell into fibrin (HE ×400). (D, E) Neoplastic HRS cells positive to CD30 (D) and CD15 (E) antibodies (immunohistochemistry ×400).

Blood testing revealed a white blood cell count of 16 × 10^9^/L, a hemoglobin level of 9.9 g/dL, a platelet count of 411 × 10^9^/L, and normal liver and renal function test results. The serum LDH level was 263 U/L. Cytological examination of the pericardial fluid indicated infiltration from lacunar cells (Reed–Sternberg), which were positive for CD15 and CD30 and indicative of HL. Histological examination of an excisional biopsy from the pericardium was characterized by lacunar cells, a variant of Reed–Sternberg cells, which exhibited positive immunostaining for CD15 and CD30, confirming the diagnosis of HL. These findings suggested the presence of the nodular sclerosing variant of the disease (Figure 3).

A head, chest, abdominal, and pelvic CT scan was performed to stage the disease, revealing cervical, mediastinal, and paracardiac lymphadenopathy. Following the initial assessment and staging, the patient was classified by Ann Arbor staging as stage IV and according to the International Prognostic Score (IPS) as intermediate-2. Combined chemotherapy with standard doxorubicin, bleomycin, vinblastine, and dacarbazine (ABVD) chemotherapy has been started, and the first cycle has been completed. The patient was discharged after 7 days from the hospital in stable clinical condition. Currently, she has undergone 5 cycles of chemotherapy and is being closely monitored for treatment response and potential adverse effects.

A PET CT was performed after 5 cycles of chemotherapy, and no abnormal findings were observed, demonstrating that she was in complete response.

## 3. Discussion

We present a patient with cardiac tamponade that occurred as the principle symptomatology of HL.

HL appears with an occurrence of 3 cases per 100,000 individuals in the Western world [1]. Approximately 20–25% of lymphoma patients exhibit cardiac involvement [6], while 9% of all cardiac tumors are associated with lymphoma [6]. However, there is limited understanding of the clinical progression and outcomes of pericardial involvement in HL.

Given the pathophysiology of cardiac tamponade, it seems that our patient exhibited a subacute presentation that gradually evolved into cardiac tamponade. Timely identification is crucial for prompt intervention, validated by clinical examination and confirmed through echocardiography, as demonstrated in our patient. Urgent pericardiocentesis is imperative to restore right ventricular function and increase venous return [6]. Various approaches exist for pericardiocentesis, with the subcostal approach being followed in our case, considered the safest [6]. In this patient, prompt drainage of pericardial fluid was performed to prevent potential complications, and the collected fluid was subjected to investigation. The diagnosis was established by histologic tissue examination, contributing to the uniqueness of this case. Despite being admitted for the assessment of chest pain and dyspnea, the patient was ultimately diagnosed to have HL.

Pericardial engagement in HL may arise from pericardial infiltration due to metastatic disease, invasion from primary cardiac tumors, benign idiopathic causes, infection, or pericarditis that may be induced by drugs or radiation [1]. Several factors may contribute to pericardial effusion in lymphoma, including lymphatic and hematogenous spread. Effusion may arise from the obstruction of lymphatic and venous drainage of pericardial fluid or occur independently of pericardial involvement.

HL may exhibit a subtle clinical onset characterized by constitutional symptoms such as weight loss, chills, night sweats, and fever. It is noteworthy that the diagnosis of HL manifests in two distinct peaks: the first occurring between ages 15 and 40 years and the second in individuals over 60 years. Interestingly, constitutional symptoms do not exclusively define the initial presentation of HL. Patients may also exhibit manifestations such as lymphadenopathy, hepatomegaly, splenomegaly, and pruritus [7, 8]. Notably, pericardial effusion is an infrequent presentation that is observed in approximately 5-6% of HL diagnoses. Most pericardial involvement in HL remains asymptomatic, identified through imaging, typically in small volumes, and rarely occurs as the primary manifestation of the disease. In a retrospective study involving 273 patients, only two cases with symptomatic pericardial effusion were reported [9]. Malignant lymphoma may originate or spread to the mediastinum, where lymphadenopathy can manifest early due to tumor growth and associated compression [10]. On the other hand, symptomatic pericardial effusion may present with dyspnea, chest pain, pleuritic thoracic pain, and peripheral edema [11].

The occurrence of cardiac tamponade as the initial presentation of HL is exceptionally rare. Hajra et al. presented a male patient with cardiac tamponade as the first presentation of HL [2]. Our patient exhibited early cardiac tamponade, although she presented with chest pain and dyspnea during the last 7 days, albeit with subtle symptoms. In a similar manner, Hajra et al. presented a young male who complained of shortness of breath for more than a week [2]. Echocardiography revealed cardiac tamponade, and immediate pericardial fluid drainage was performed [2]. Othman et al. also reported a case of cardiac tamponade in a young male patient with an undiagnosed HL [3]. He was characterized by a subacute presentation, and emergency pericardiocentesis was performed. However, he was complicated by disseminated nontyphoidal salmonellosis, which was not an issue in our patient [3]. Similar management was reported by Chango Azanza and Vredenburgh who described a young male who complained of worsening exertional dyspnea and orthopnea for more than a month [12]. The echocardiogram showed a hemodynamically significant large pericardial effusion, which was treated with pericardiocentesis and pericardial drain. After a CT-guided biopsy of the mediastinal mass, the diagnosis of nodular sclerosis HL was confirmed [12]. All patients were young individuals who presented with symptoms lasting more than a week. In all cases, cardiac tamponade was the first manifestation of HL. Therefore, these cases highlight the need for early detection and timely intervention.

Standard chemotherapy with the combined modality of ABVD is recommended in early stages of the disease [13]. Our patient was initiated on ABVD and was discharged in a stable clinical condition. The same treatment scheme was followed in the cases presented by Hajra et al. Azanza et al., and Othman et al. The specific HL subtype in our case is nodular sclerosis, which is frequently associated with a mediastinal mass [14]. The association between pericardial involvement in nodular sclerosis HL has been reported in several studies [2, 9, 15–17].

## 4. Conclusion

Malignant cardiac tamponade is an uncommon complication associated with HL, and its presence constitutes a critical and life-threatening emergency. The case gains particular interest due to the initial presentation of cardiac tamponade in a young female patient with an undiagnosed HL. The traditional signs and symptoms of tamponade often lack sensitivity for an accurate diagnosis. Any pericardial effusion significant for tamponade requires immediate drainage and fluid analysis for thorough investigation. The rapidity of fluid accumulation in the pericardial cavity influences the urgency of cardiac tamponade presentation, suggesting the potential for more aggressive subsequent episodes of pericardial effusion leading to tamponade. Therefore, clinicians should maintain heightened awareness in such cases; prompt identification and timely intervention are crucial in effectively addressing these complex situations.

## Data Availability

The data used to support the findings of this study are available from the corresponding author on request.
